# Structural dependency of polymer dynamics by means of small-angle X-ray photon correlation spectroscopy and wide-angle X-ray scattering on the D2AM beamline

**DOI:** 10.1107/S1600577525001626

**Published:** 2025-04-01

**Authors:** Grégory Stoclet, Duncan Schwaller, Romain Garlet, Frédéric Livet, Gilbert A. Chahine, Nils Blanc, Maxime Dupraz

**Affiliations:** a Univ. Lille, CNRS, INRAE, Centrale Lille, Unite Materiaux et Transformations, 59655Lille, France; b ESRF – The European Synchrotron, 71 Avenue des Martyrs, 38043Grenoble, France; c SIMaP, Grenoble INP, CNRS Université Grenoble Alpes 38000Grenoble France; d Université Grenoble Alpes CNRS, Grenoble INP, Institut Néel 38000Grenoble France; Advanced Photon Source, USA

**Keywords:** X-ray photon correlation spectroscopy (XPCS), polymer dynamics, crystallinity, coherence, macromolecular orientation

## Abstract

X-ray photon correlation spectroscopy (XPCS) has become a pivotal technique for exploring nanoscale dynamic phenomena across various materials, facilitated by advancements in synchrotron radiation sources and beamline upgrades. The recent Extremely Brilliant Source (EBS) upgrade at the European Synchrotron Radiation Facility (ESRF) in Grenoble, France, has notably improved brilliance and coherence length, thereby enhancing the capabilities of XPCS and related techniques. Here, we present a dedicated setup on the D2AM beamline at the ESRF, enabling simultaneous XPCS and wide-angle X-ray scattering measurements. The setup developed and its performance are detailed in the first part. Then, the XPCS capabilities are evaluated by studying polymer-based materials, with particular attention to the effects of temperature, crystallinity and macromolecular orientation on polymer dynamics. The study on the influence of temperature revealed that XPCS in the case of entangled polymers is an efficient technique to probe the dynamics of the macromolecular network, complementary to classical spectroscopy techniques. In addition, *in situ* measurements during the polymer crystallization revealed that increased crystallinity slows down macromolecular dynamics. Conversely, studies on stretched samples indicate that macromolecular orientation accelerates these dynamics. This work represents a novel investigation into the effect of crystallinity on macromolecular dynamics using XPCS, opening new avenues for research in polymer science.

## Abstract 2

The BM02/D2AM beamline at the ESRF is shown to be suitable for performing X-ray photon correlation spectroscopy experiments with a flux that prevents samples’ irradiation effects, features particularly suitable for polymer-based materials.

## 1. Introduction

Over the years, X-ray photon correlation spectroscopy (XPCS) has emerged as a powerful technique for probing the dynamic behaviour of materials at the nanometre scale. Thanks to this technique, a broad range of phenomenon and dynamics can be studied in a characteristic time range currently varying from hours to microseconds using fourth-generation synchrotrons, and down to the femtosecond thanks to the new free-electron laser sources (Lehmkühler *et al.*, 2021 ▸) (FELs). The versatility of XPCS has been used to study various phenomenon on various types of materials (Sandy *et al.*, 2018 ▸) such as metallic glasses (Ruta *et al.*, 2017 ▸), nanoparticles dispersed in solution (Liu *et al.*, 2021 ▸), proteins (Bin *et al.*, 2023 ▸), ice (Li *et al.*, 2023 ▸) and polymers (Nogales *et al.*, 2016 ▸; Genix & Oberdisse, 2015 ▸). Notably, XPCS measurements can be performed *in situ* or *operando*, often in combination with complementary techniques like wide-angle X-ray scattering (WAXS), further enhancing its applicability. The adoption of XPCS has been driven by significant advancements in synchrotron radiation sources and beamline upgrades. In this context, the recent upgrade of the European Synchrotron Radiation Facility (ESRF) in Grenoble, France, known as the Extremely Brilliant Source (EBS) upgrade, has yielded significant advancements, notably regarding the brilliance and the coherence length of the X-ray beam. The improvement in brilliance is directly linked to a substantial reduction in the horizontal emittance of the source, primarily benefiting insertion device (ID) beamlines. On the other hand, the enhancement in coherence length extends its positive impact to bending magnet (BM) beamlines as well. For example, at the BM02-D2AM beamline (ESRF, France), the source size experienced a notable reduction by a factor of two in the horizontal direction and a factor of three in the vertical direction (Chahine *et al.*, 2019 ▸), resulting in a remarkable increase in transverse coherence length, opening new avenues for the use of coherence on the beamline. The ESRF upgrade has not only enhanced beamline capabilities but has also broadened the development of characterization techniques that leverage a coherent X-ray beam, including XPCS, ptychography and Bragg coherent diffraction imaging. These advancements open up exciting prospects for the scientific community. To capitalize on the ESRF upgrade, a dedicated setup has been devised on the D2AM beamline (ESRF, France), enabling simultaneous XPCS measurements with WAXS measurements.

This work first presents the setup and its technical performance. Subsequently, the XPCS capabilities are evaluated through the study of polymer-based materials, focusing on the effects of temperature, crystallinity and macromolecular orientation on polymer dynamics. While previous studies have explored XPCS in polymers, many aspects remain underexplored. For instance, some studies have focused on the glass transition process in low-molecular-weight polystyrene (Guo *et al.*, 2009 ▸) or atactic polystyrene melts (Hoshino *et al.*, 2013 ▸). A significant discovery from both research groups is the observation of a transition from diffusive motion to hyperdiffusive behaviour when the temperature surpasses 1.1 to 1.25 times the glass transition temperature of the material. This transition occurs as the polymer shifts from its glassy to its rubbery state. Authors also reported a steady decrease of the relaxation time (τ) with the increase of the temperature, despite the relatively narrow temperature range studied, which was centred around the glass transition. Other studies have focused on investigating how macromolecular orientation and filler-matrix interactions influence the dynamics in elastomers (Ehrburger-Dolle *et al.*, 2012 ▸; Ehrburger-Dolle *et al.*, 2019 ▸). Interestingly, they found that macromolecular orientation leads to anisotropic and heterogenous dynamics that they tentatively linked to the structure of the stretched cross-linked macromolecular network. However, to the best of our knowledge, the influence of crystallinity on macromolecular dynamics using XPCS remains unexplored. This study addresses this gap, providing novel insights into polymer dynamics under varying conditions.

## 2. Materials and methods

### 2.1. Materials and sample preparation

The beamline capacities were first evaluated using a 1 mm-thick compacted powder of fumed silica (Aerosil200), chosen for its ability to provide stable and strong X-ray scattering over a wide range of wavevectors. For the polymer-based composites, polylactide (PLA) was selected as the polymeric matrix. This polymer is a widely used bio-based polyester, exhibiting properties comparable with those of polyethyl­ene terephthalate (PET). Within this matrix, silica nanoparticles (fumed silica S5130 from Sigma-Aldrich) were dispersed and served as tracers. These are spherical nanoparticles with an average diameter of 200–300 nm.

In this study, two different grades of PLA were used. The first one is PLA grade 4060D (called PLA60D) which contains about 10% of D-isomer units, rendering it amorphous and unable to crystallize. The second grade used is PLA grade 4043D (PLA43D), which contains about 2% of D-isomer units. This PLA grade is able to crystallize and exhibits slow crystallization kinetics (Stoclet *et al.*, 2007 ▸). The main characteristics of the PLA grades are summarized in Table 1 ▸.

The nanocomposites were prepared by extrusion in the molten state using an Xplore micro extruder (DSM Research). Prior to their extrusion, PLA pellets were dried overnight under vacuum at 60°C to prevent hydrolytic degradation during the processing. Then, 13.5 g of PLA and 1.5 g of silica (corresponding to a weight fraction of nanoparticles of 10 wt%,*i.e.* a volume fraction of 5 v/v%) were mixed during 3 min at 190°C at a screw speed of 50 r.p.m. The extruded pellets were then dried during 24 h at 60°C under vacuum. Thin films, approximately 200 µm thick, were subsequently produced through compression moulding. This process involved compressing the dried pellets for 2 min at 180°C under 50 bar of pressure.

### 2.2. Sample’s characterization

Prior to measurements, viscoelastic properties, thermal properties and structure of the samples were characterized. Viscoelastic properties of the materials were determined by means of dynamic mechanical analysis (DMA) experiments in tensile mode. The measurements were carried out on an RSA3 apparatus (TA Instruments) on 0.2 mm-thick rectangular specimens (15 mm × 5 mm) in the 25–130°C temperature range at a frequency of 1 Hz. Thermal properties were determined through differential scanning calorimetry (DSC) experiments performed on a DSC Q20 apparatus (TA Instruments). The temperature range for the DSC analysis was 25°C to 180°C, with heating and cooling rates of 10°C min^−1^. Temperature and heat flow calibrations were performed using a high-purity indium standard following established protocols. Experiments were conducted under nitro­gen flow with sample weights of approximately 5 mg placed in sealed alumina pans. The structural characterization of the prepared samples was performed using small-angle X-ray scattering (SAXS) and WAXS measurements. These experiments aimed to determine the nature of the samples (amorphous or semi-crystalline) and to evaluate the dispersion quality of the silica nanoparticles within the polymer matrix. SAXS and WAXS data were collected using a Xeuss 2.0 SAXS/WAXS system (Xenocs, France) operating at a wavelength of λ = 1.54 Å.

The WAXS analysis confirmed that both PLA60D and PLA43D nanocomposites were amorphous after processing [see Fig. S1(*a*) of the supporting information]. Meanwhile the SAXS analysis [Fig. S1(*b*) of the supporting information] indicated that both materials exhibit the same scattering, suggesting a good dispersion of the silica nanoparticles into the polymer matrix. This good dispersion degree is supported by (i) the complete transparency of the films to the naked eye and (ii) the SEM micrographs depicted in Fig. S2 of the supporting information.

## 3. Experimental

### 3.1. Beamline and setup description

**Figure 1:**
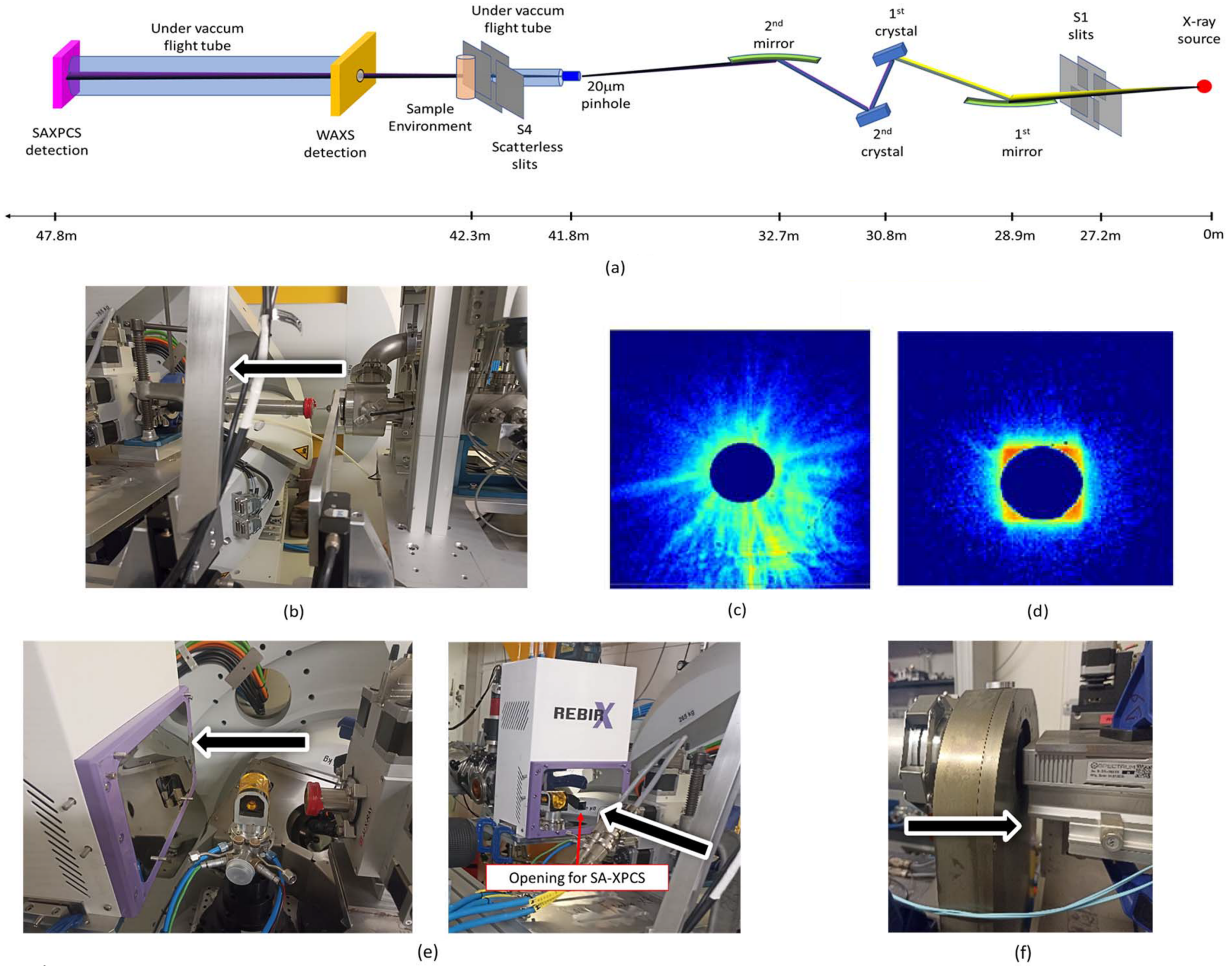
(*a*) Schematic description of the beamline optics and of the setup used for the simultaneous SA-XPCS/WAXS experiments. Photographs of (*b*) the pinhole used, and patterns of the beam (*c*) after the pinhole and (*d*) after the S4 guard lists. (*e*) Sample environment and WAXS open for SA-XPCS detection and (*f*) the SA-XPCS detection part. The black arrows symbolize the beam direction.

The experiments were performed on the BM02-D2AM beamline at the ESRF at a beam energy of 8 keV (wavelength: 1.55 Å). This energy was selected to maximize the coherent flux on the sample and optimize the size of the speckles. The experimental setup is illustrated in Fig. 1 ▸.

#### 3.1.1. Beamline optics

The beamline optics consist of:

(i) First mirror (M1): located 29 m downstream of the source, this mirror provides vertical reflection of the polychromatic incident beam to suppress the λ/3 harmonics and produce a parallel polychromatic beam.

(ii) Double-crystal monochromator (DCM): positioned approximately 2 m downstream of M1, the DCM uses two Si(111) crystals. The first crystal monochromatizes the beam with an energy resolution Δ*E*/*E* of 1.4 × 10^−4^. The second crystal, mounted on a sophisticated mechanical system, allows for precise adjustments, including tilting and bending, to horizontally focus the monochromated beam.

(iii) Second mirror (M2): approximately 2 m downstream of the second crystal, M2 reflects the horizontally monochromated beam downwards and restores it to the horizontal axis defined by the source. Like M1, M2 is equipped with benders that induce an ellipsoidal curvature to vertically focus the beam.

Further technical details on the beamline are given by Chahine *et al.* (2019 ▸).

As illustrated in Table S2 of the supporting information, this optical configuration delivers a flux of 1.5 × 10^11^ photons s^−1^ in a focused beam of ∼35 µm (V) × 35 µm (H) when the primary slits (S1) are opened to 5 mm (V) × 10 mm (H). In the conditions of the experiment, with the primary slits set to 0.5 mm (V) × 0.5 mm (H), the flux was reduced to approximately ∼8.25 × 10^8^ photons s^−1^.

#### 3.1.2. Beam focusing and conditioning

The monochromatic beam was focused onto a 20 µm Pt:Ir (95:5) pinhole located 50 cm upstream of the sample. This step further reduced the total flux by approximately a factor of five, resulting in a final flux *I*_0_ of 1.7 × 10^8^ photons s^−1^ (Table S2 of the supporting information). Guard slits were placed 15 cm upstream of the sample to eliminate parasitic scattering originating from the pinhole.

#### 3.1.3. Coherence of the beam

As mentioned earlier, the DCM Si(111) provides an energy resolution of Δλ/λ = 1.4 × 10^−4^. At 8 keV, this gives a longitudinal coherence of
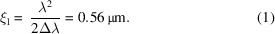
This value is large enough to ensure that the contrast will not decay monotonically as a function of *q* in the *q* range considered for the experiment. Regarding the transverse coherence, the very small source size [8.5 µm (V) × 54 µm (H)] ensures relatively large transverse coherence lengths at the entrance of the S1 slits, located at 27.2 m from the source. Using the formula
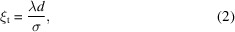
where *d* is the distance from the source and σ the source size, gives values of 496 µm and 78 µm for the vertical and horizontal coherence lengths, respectively. It comes naturally that these values are much smaller for the focused beam after the optics.

#### 3.1.4. Sample environment

The sample was mounted in transmission geometry within a dedicated ceramic furnace provided by the ESRF Sample Environment Group. The sample was secured using a ceramic screw with a 3 mm opening to allow X-ray beam passage. The setup was shielded by a Kapton dome to minimize temperature fluctuations, a critical factor for XPCS measurements. The furnace’s PID controller was finely tuned to operate in the room temperature to 200°C range, ensuring rapid temperature stabilization and minimizing temperature overshoots. Additional details about the furnace setup are provided in Section S4 of the supporting information.

#### 3.1.5. Detector configuration

The WAXS signal was captured using a Si-based 2D WOS (WAXS Open for SAXS, IMXPAD) photon-counting detector with dimensions 1120 × 600 pixels and a pixel size of 130 µm. The detector was positioned 14 cm downstream of the sample. The WOS detector features a 19 mm opening in its active area, allowing simultaneous detection of the WAXS signal and transmission of the primary beam for SAXS measurements. The SAXS signal was recorded using a Lambda X-spectrum Si-based photon-counting detector (Lbd) with dimensions 516 × 516 pixels and a pixel size of 55 µm. The Lbd detector was positioned ∼5.5 m downstream the sample. This configuration enables simultaneous small-angle X-ray photon correlation spectroscopy (SAXPCS) and WAXS measurements, which is critical for monitoring the crystallization processes in the samples. For simplicity, SAXPCS is referred to as XPCS throughout the manuscript.

## 4. Data processing and analysis

### 4.1. XPCS data processing

**Figure 2:**
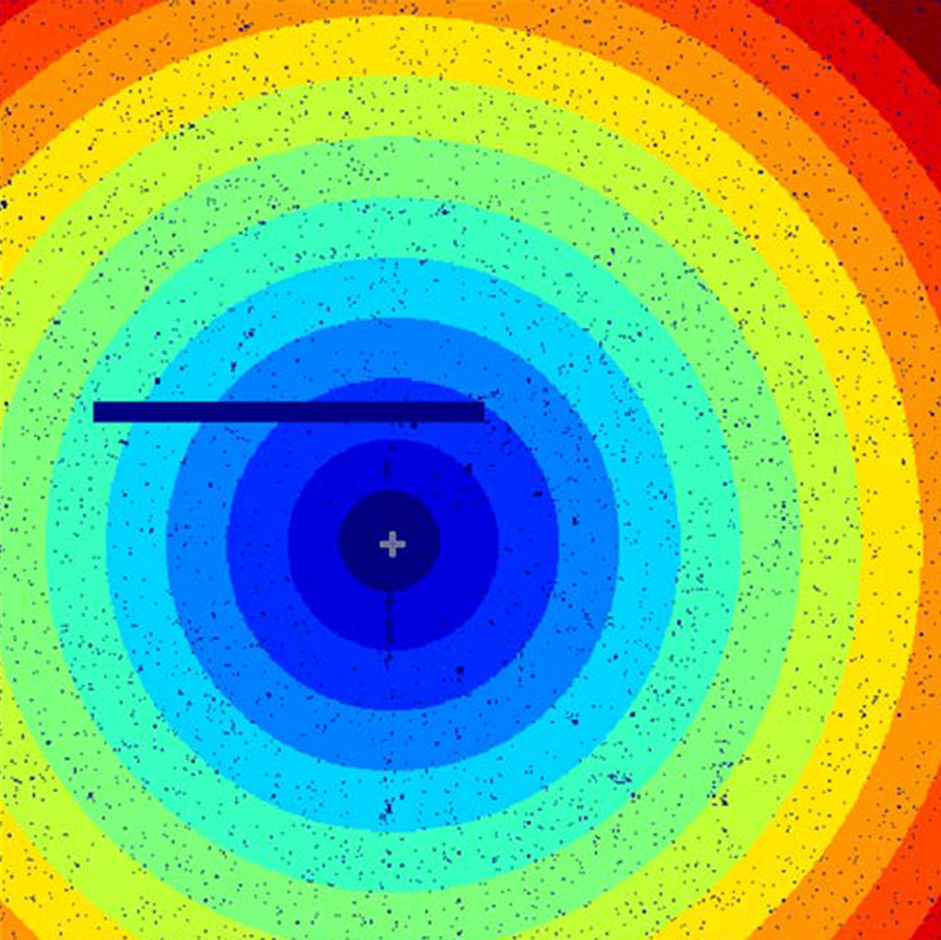
Scheme of the analysed zones (bins) used for the correlation functions calculations.

Prior to processing, a mask was applied to the recorded patterns to remove bad pixels and account for the lambda detector gaps. Then, a scattering vector map *q*_map_ was generated using the *pysimplemask* package (https://github.com/AdvancedPhotonSource/pySimpleMask) to define integration bins for calculating relaxation times as a function of the scattering vector *q*. Basically the patterns were divided into 12 circular bins, as illustrated in Fig. 2 ▸, covering a *q* range from 1.2 × 10^−3^ to 10^−2^ Å^−1^.

The autocorrelation function *g*_2_(*t*), also called the intensity–intensity correlation function, as well as the two-time correlation function were computed using the *boostcor* package (https://github.com/AdvancedPhotonSource/boost_corr.git). The *g*_2_(*t*) function represents the correlation between the intensity fluctuations of the scattered X-rays at a given time *t* and the intensity fluctuations at a later time *t* + τ, where τ represents the time delay. In other words, the autocorrelation function in XPCS experiments serves as a tool for quantifying and analysing the temporal correlations in the intensity fluctuations of scattered X-rays, in order to determine the dynamic behaviour of materials at the nanoscale. Mathematically, *g*_2_(*t*) is calculated for each of the circular bin and can be expressed as
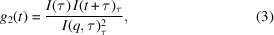
with *q* the scattering vector (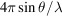) and 2θ the scattering angle.

The two-time correlation function is particularly well suited for studying equilibrium and non-equilibrium relaxation phenoma (Bikondoa, 2017 ▸). This function facilitates the visualization of temporal variations in correlations and/or dynamic heterogeneity by replacing the time-averaged correlation function with the instantaneous intensity correlation function, also known as the two-time correlation function. According to Fluerasu *et al.* (2005 ▸), the two-time correlation function is defined as the covariance of the scattered intensity,
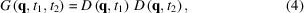
where *t*_1_ and *t*_2_ correspond to the time elapsed from the beginning of the measurement (*t* = 0) and *D*(**q**,*t*) is the normalized intensity fluctuation defined as
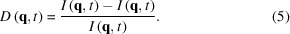
As for *g*_2_(*t*), *G*(*q*, *t*_1_, *t*_2_) functions were calculated on different regions of interest (ROIs) corresponding to the 12 circular bins determined before (see Fig. 2 ▸).

Data visualization and relaxation times (τ) calculations were performed using the *pyXPCSviewer* tool (Chu *et al.*, 2022 ▸). In this work, relaxation times τ were extracted by fitting the autocorrelation curves with a single exponential decay model, as represented by
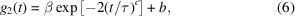
where β corresponds to the contrast, *c* the stretch/compression ratio of the exponential and *b* the baseline level (generally equal to 1 when a complete decorrelation is achieved).

### 4.2. WAXS data processing

For the WAXS analysis, the sample to detector distance has been calibrated using reference powders of silver behenate (AgBeh) and lanthanum hexaboride (LaB_6_). Prior to processing, a mask was applied to the patterns to remove hot and dead pixels and the gaps between the detector modules. Radial integrations [*I* = *f*(2θ)] as well as azimuthal integration [*I* = *f*(Φ)] were performed using homemade Python-based scripts using the *pyFAI* Python library (Ashiotis *et al.*, 2015 ▸).

## 5. XPCS measurements and performance of the beamline

**Figure 3:**
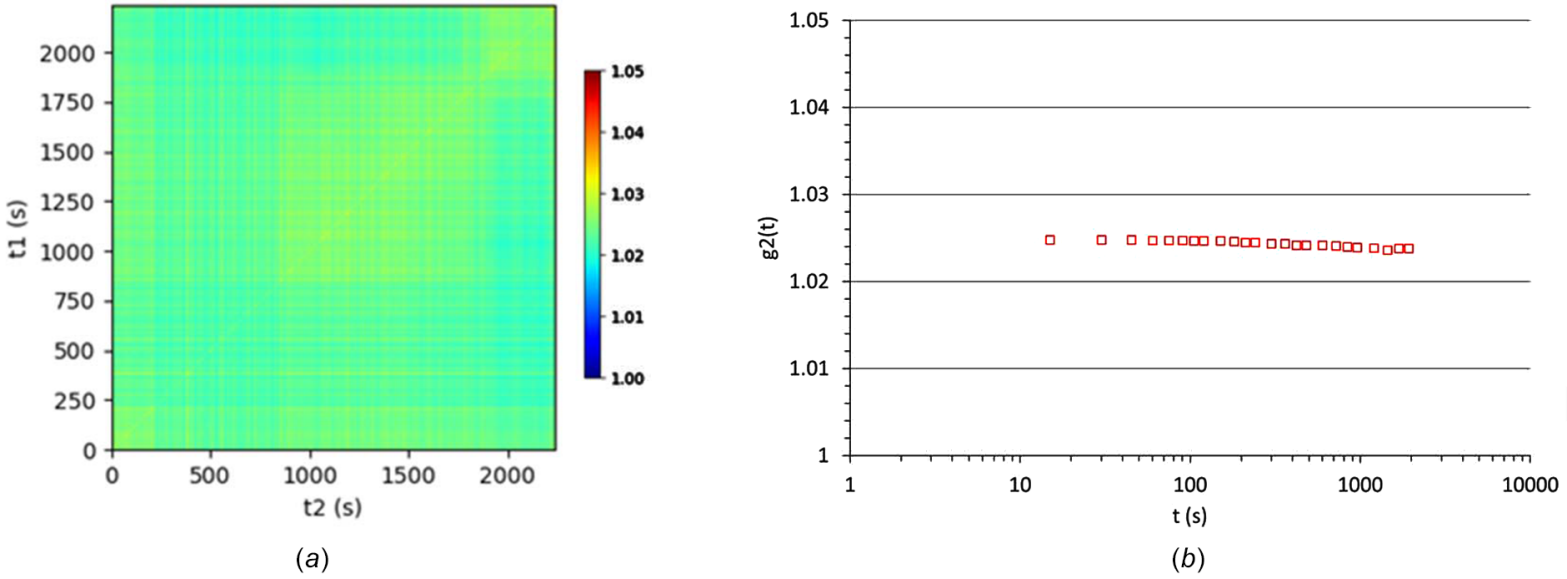
(*a*) Two-time correlation function and (*b*) autocorrelation function calculated at *q* = 0.002 Å^−1^ for the reference aerogel sample [measurements performed with a primary slits (S1) opening of 0.5 mm × 0.5 mm].

To evaluate the optimal conditions for XPCS measurements and assess the stability of the experimental setup, the aerogel sample was employed as a reference. The acquisitions consisted of a sequence of 100 frames with an acquisition time of 30 s per frame. As depicted in Fig. 3 ▸, unlike the PLA samples, the aerogel sample remains static throughout the measurement interval, with no evidence of decorrelation observed in *g*_2_(*t*).

It is worth noting that these measurements were conducted with various slit openings and exposure times, yielding comparable results. Ensuring a high stability of the setup is of utmost importance for accurately measuring the relaxation times within the samples. This use of a static sample allows for an accurate characterization of the degree of coherence (β). More importantly, β strongly depends on the opening of the primary slits, as discussed in extensive detail by Livet (2007 ▸). As shown in Fig. S3 of the supporting information, the experimental degree of coherence is consistent with the theoretical calculations. Additionally, owing to the small source size available on the BM02-D2AM beamline (8 µm × 54 µm), relatively high degrees of coherence can be achieved (Raimondi *et al.*, 2023 ▸).

This implies that, despite the lower brilliance offered by a bending magnet beamline such as D2AM compared with an undulator beamline, coherence applications including XPCS measurements are feasible. In particular, D2AM beamline is perfectly suitable for the measurements of dynamics dealing with irradiation-sensitive samples exhibiting slow to medium relaxation times (*i.e.* down to the second).

While Table 2 ▸ might initially suggest that a smaller opening of the primary slits might yield more favourable conditions for XPCS measurements, the high degree of coherence achieved comes at the expense of the flux. Therefore, a relevant metric to select the optimal measurement conditions is the coherent flux, *i.e.* the product of the incoming photon flux times the degree of coherence (β*I*). Calculating this quantity, as a function of the slit opening, reveals that β*I* reaches a maximum of 4.2 × 10^6^ photons s^−1^ for a slit opening of 0.5 mm × 0.5 mm. Further opening of the slits has only a marginal effect in terms of gain on the coherent flux. In addition, β values below 1% leads to excessive speckle averaging which is undesirable for XPCS measurements. Based on these results, an opening of 0.5 mm × 0.5 mm for the S1 slits was selected for XPCS measurements. With such experimental conditions, the coherent flux is admittedly much lower than the values offered by undulator beamlines, which can be orders of magnitude higher, particularly in fourth-generation synchrotrons. However, this setup is perfectly suitable for samples sensitive to radiation damage and with relatively slow dynamics, such as the ones used in this work.

## 6. Application to the measurement of polymer dynamics

### 6.1. Setup and measurement procedure

Macromolecular dynamics were studied at different temperatures during isothermal measurements. Each measurement lasted approximately one hour, consisting of continuous image recordings from 300 to 3000 images with an acquisition time varying from 10 s to 1 s, respectively. As mentioned earlier, WAXS data were simultaneously collected with the SA-XPCS signal to monitor structural changes. Prior to each measurement, a five-minute delay was implemented once the desired temperature was reached, ensuring thermal equilibrium within the sample. The samples analysed were 3 mm × 3 mm squares cut from thin films and placed in a home-made ceramic furnace (details in Section S4 of the supporting information). This furnace effectively shielded the sample from its environment and enabled precise temperature regulation, *i.e.* ±0.1°C. The total duration of one hour was chosen to ensure complete decorrelation within the sample.

### 6.2. Influence of temperature on the polymer dynamics

**Figure 4:**
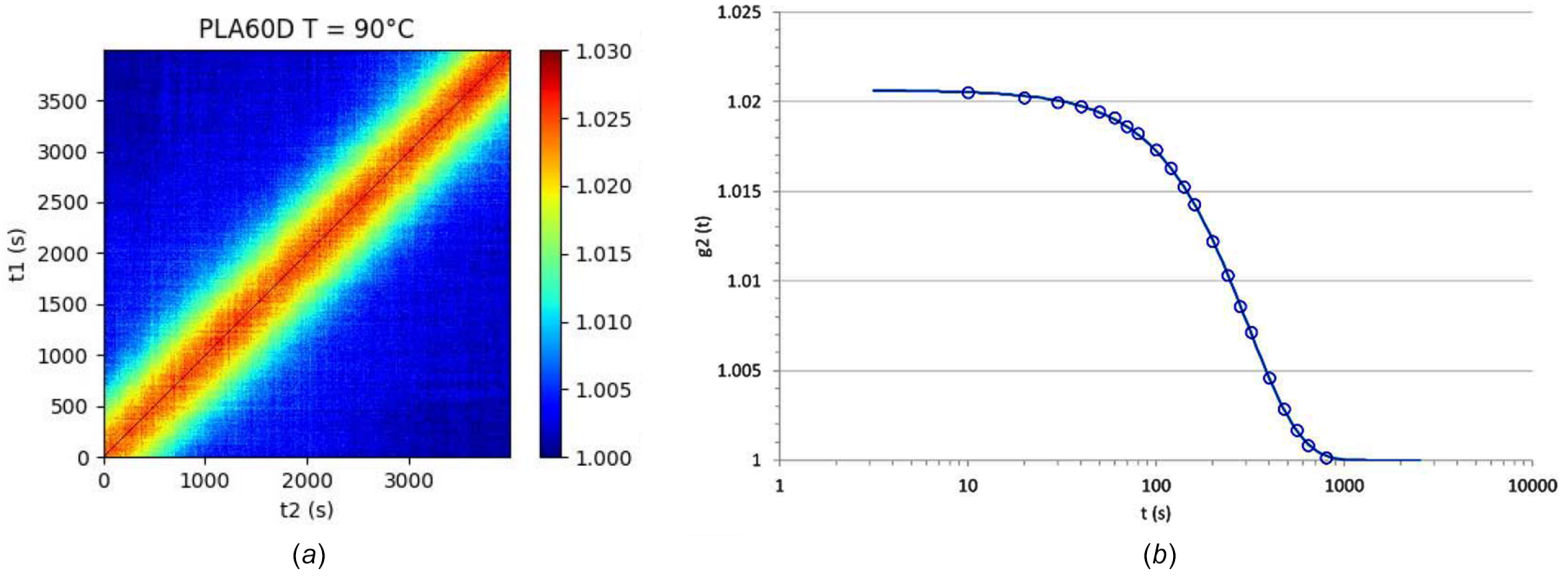
(*a*) Two-time correlation function and (*b*) autocorrelation function calculated at *q* = 0.004 Å^−1^ measured for PLA60D at 90°C.

The influence of temperature on macromolecular dynamics has been explored in previous studies, particularly through XPCS investigations on polystyrene (Guo *et al.*, 2009 ▸; Hoshino *et al.*, 2013 ▸). It was found that (i) the transition from the glassy to the rubbery state of the polymer is characterized by a transition from diffusive motions to a hyperdiffusive behaviour accompanied by a steady decrease in the measured relaxation time. Nevertheless, it is worth noting that in those studies the polymer had a molecular weight below its mass between entanglements (*M*_e_) which means that in these materials the macromolecules do not interpenetrate nor form an entangled, and thus constrained, macromolecular network. In contrast, the PLA samples used in this study, as well as most plastics, are entangled polymers with a molecular weight larger than *M*_e_ (approximately 6000 g mol^−1^) (Stoclet *et al.*, 2014 ▸). As a result, the macromolecules form a highly entangled network in our materials, which have a molecular weight of 60–70 kg mol^−1^. To examine the temperature’s effect on dynamics, isothermal measurements were performed at temperatures ranging from 30°C to 150°C. A representative example of results at *T* = 90°C is shown in Fig. 4 ▸.

**Figure 5:**
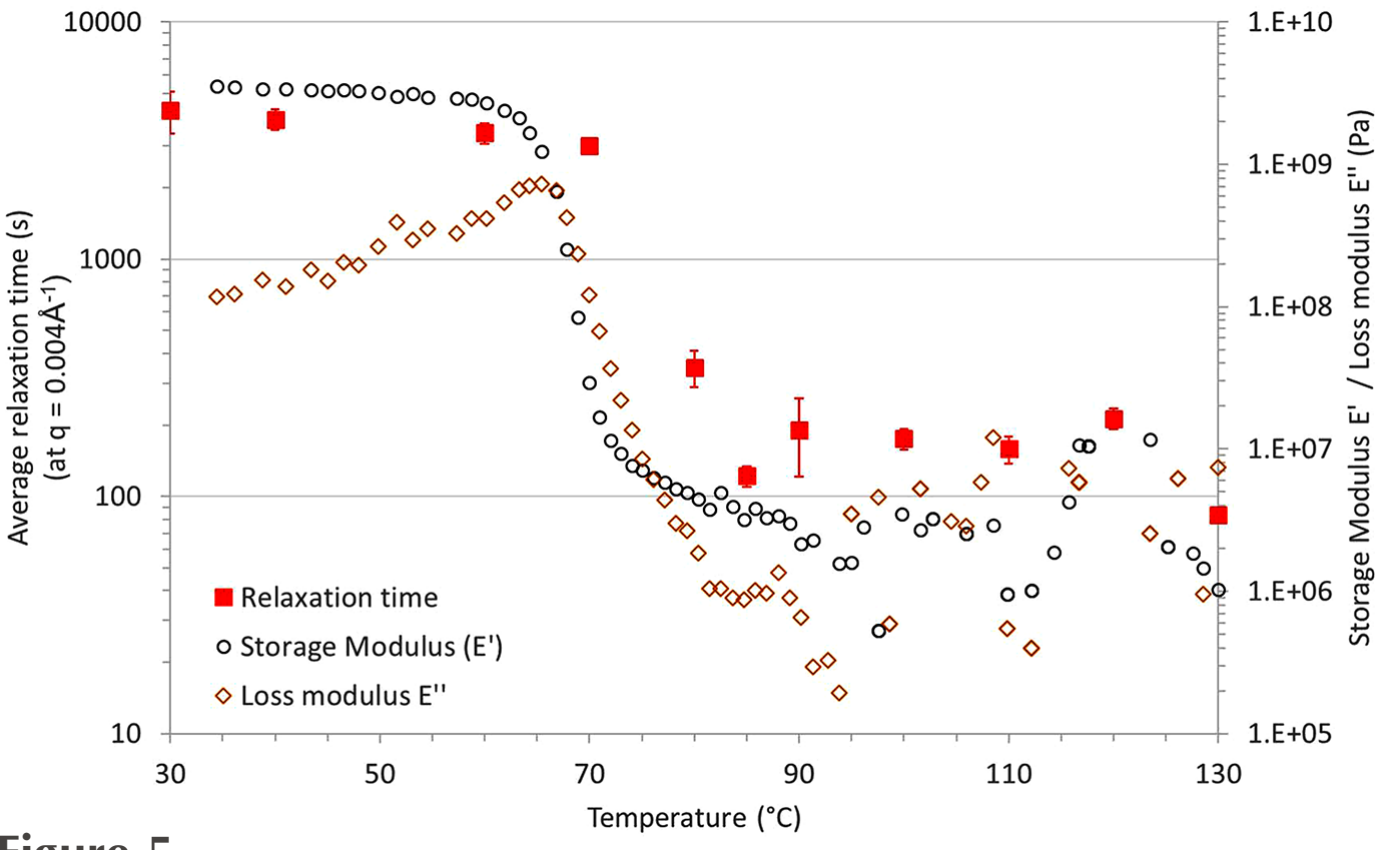
Evolution of the relaxation time τ, storage modulus *E*′ and loss modulus *E*′′ as a function of PLA in the case of PLA60 filled with 10 wt% of silica.

As revealed by the two-time correlation function [Fig. 4 ▸(*a*)], the dynamics within the material remained stationary throughout the entire measurement at *T* = 90°C, indicating that the sample reached an equilibrium state. The autocorrelation function, depicted in Fig. 4 ▸(*b*), exhibits a single exponential decay with a characteristic relaxation time *t* ≃ 200 s. This behaviour is similar to the ones reported by Guo *et al.* (2009 ▸) and Hoshino *et al.* (2013 ▸). Based on the measurements carried out over the temperature range 30–130°C, the influence of the temperature on the average relaxation time can be evaluated. This is illustrated in Fig. 5 ▸, which depicts the evolution of τ alongside the storage modulus (*E*′) and loss modulus (*E*′′) as functions of temperature. (The storage and loss modulus, *E*′ and *E*′′, respectively, were measured using DMA.)

**Figure 6:**
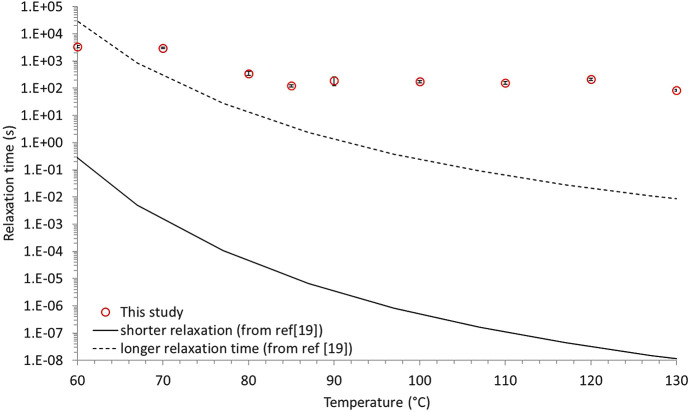
Evolution with temperature of the relaxation times measured by means of XPCS (this work) and by means of broadband dielectric spectroscopy [from Mierzwa *et al.* (2002 ▸)].

Regarding the average relaxation time τ, two regions can be distinguished: below 80°C, τ remains relatively constant around 3000 s; above 80°C, τ sharply decreases and stabilizes around 200 s. In parallel, the storage modulus *E*′ shows a sharp decrease from approximately 3 GPa to roughly 4 MPa around 70–80°C, reflecting the transition from a rigid (glassy) state to a soft (rubbery) one, which is characteristic of the glass transition of the polymer. This change in mechanical properties coincides well with the variations of the relaxation time. The loss modulus *E*′′ exhibits two plateau regions, one below ∼60°C and another above ∼80°C, with a broad peak observed between them. This drop is associated with energy dissipation due to chain friction during the glass transition, marking the end of the transition. The endpoint, where all the macromolecular mobilities are activated within the polymer, coincides well with the onset of the ‘short relaxation time plateau’. The decrease in the relaxation time when the material transitions to its rubbery state has been reported in other studies. These studies, whether using XPCS (Genix & Oberdisse, 2015 ▸) or other techniques (Mierzwa *et al.*, 2002 ▸; Paluch *et al.*, 2000 ▸; Williams *et al.*, 1955 ▸), attribute this decrease to thermal activation. Notably, Mierzwa and co-workers studied the effect of molecular weight on the relaxation dynamics of this polymer using broadband dielectric spectroscopy (Williams *et al.*, 1955 ▸). They found that above the α relaxation process, *i.e.* above the glass transition, the relaxation time follows a Vogel–Fulcher–Tamman law, as for many other polymers. In their study, they evaluated the influence of temperature on (i) the shorter relaxation time which corresponds to local molecular motions and (ii) the longest relaxation time which is ascribed to cooperative movements of the macromolecules. For the sake of comparison, Fig. 6 ▸ depicts the evolution of the relaxation time τ with the temperature, for the measurements of this work and the relaxation times determined by Mierzwa *et al.* (2002 ▸).

Interestingly the relaxations times measured in this study are longer than both the shortest and longest relaxation times reported by Mierzwa *et al.* (2002 ▸). Moreover, while the relaxation times from their study decrease steadily with increasing temperature, our XPCS measurements show a constant relaxation time above the glass transition temperature of PLA.

The discrepancy between the relaxation time measured in our study and the ones determined by broadband dielectric spectroscopy can be explained by the different scales of motions being analysed. Our measurements correspond to large displacements at the nanometre scale (measured at low *q*) while dielectric spectroscopy focuses on short ranges motions, at the atomic scale, which are consequently faster. On the other hand, the fact that in our study τ is roughly constant above *T*_g_ tends to show that the dynamics measured are not reflective of local macromolecular motions but rather depict the dynamics of the entangled macromolecular network. In other words, XPCS measurements capture the dynamics of the silica nanoparticles trapped within an entangled constraining network, rather than the motions of the macromolecules themselves. This interpretation also rationalizes the finding of a decreasing relaxation time with temperature given by Chahine *et al.* (2019 ▸) and Guo *et al.* (2009 ▸), where the polymer was not entangled but rather consists of ‘independent’ macromolecules. In such a case, viscoelastic properties measurement would not have given a *E*′ plateau but rather a steady decrease in the *E*′ value.

### 6.3. Influence of crystallinity on the dynamics

**Figure 7:**
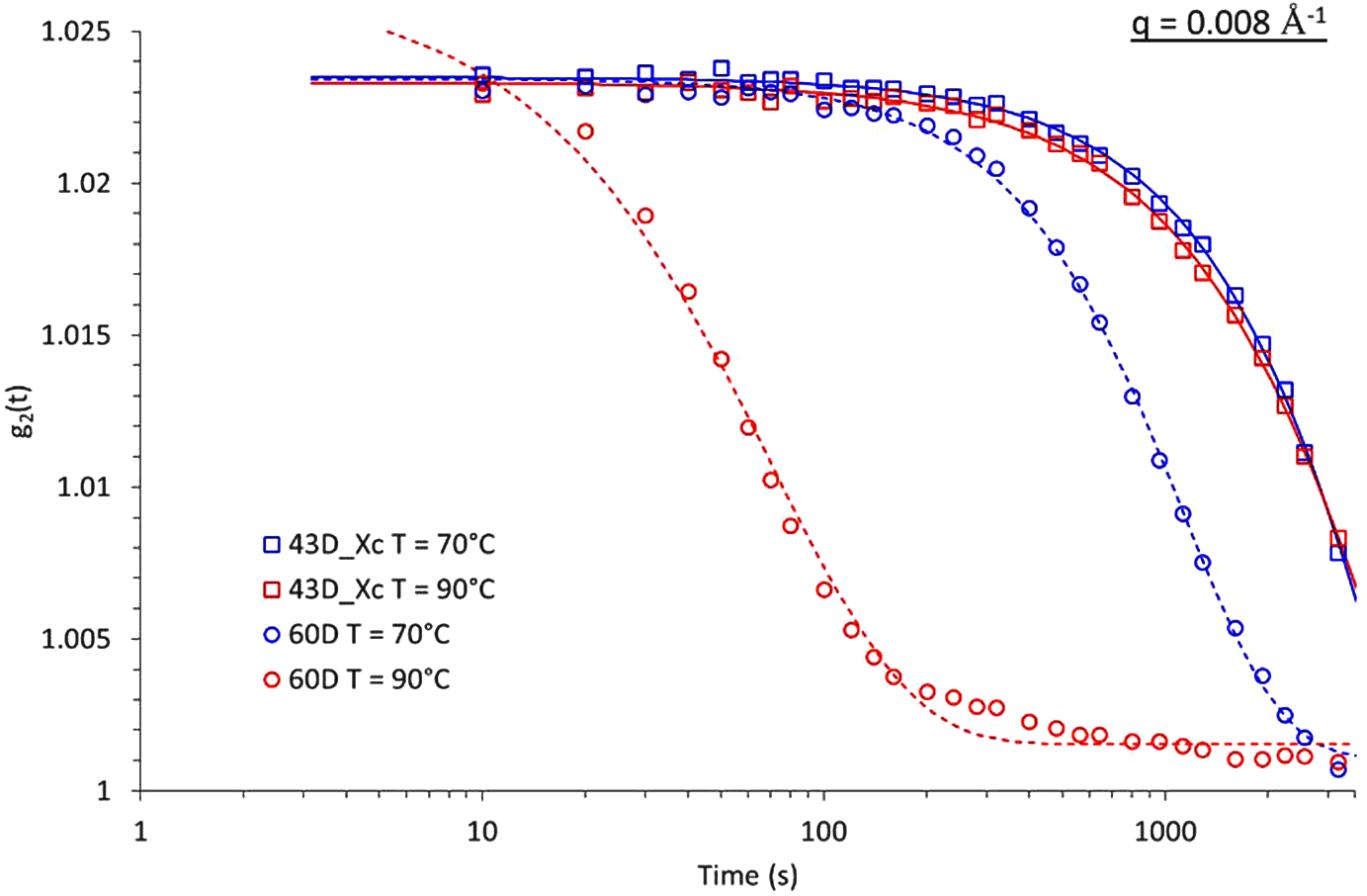
Autocorrelation functions and fitted curves measured for an amorphous PLA (blue curves) and a semi-crystalline PLA (red curves, *X*_c_ ≃ 35%) measured at *T* = 70°C for *q* = 0.008 Å^−1^.

The influence of crystallinity on the polymer dynamics has been widely studied, primarily using spectroscopy techniques such as nuclear magnetic resonance and dielectric spectroscopy. However, to the best of our knowledge, no study has yet investigated this topic using XPCS. In this work, we aim to explore the potential effects of crystallinity on the macromolecular dynamics of the polymer matrix by comparing the dynamics of a fully amorphous PLA (PLA60D) with a semi-crystalline PLA (PLA43D, with a crystallinity degree of 35%) at two temperatures, 70°C and 90°C. For these measurements, the semi-crystalline PLA43D was crystallized from the melt at 120°C during 2 h to reach the maximum crystallinity degree achievable by the material. Fig. 7 ▸ depicts the autocorrelation functions calculated from the experimental data for both amorphous and semi-crystalline PLA.

It clearly appears that, while a complete decorrelation is observed for the amorphous sample, only a partial decorrelation is observed for the semi-crystalline sample over the same time range. Additionally, while the relaxation times for the amorphous sample are around 1750 s at 70°C and 135 s at 90°C, they are significantly longer for the semi-crystalline sample, at approximately 6200 s and 5800 s, respectively. This clearly indicates that the presence of crystals significantly impacts the macromolecular network dynamics, resulting in increased relaxation times. Similar behaviour has already been reported by Delpouve *et al.* (2014 ▸). Furthermore, the temperature dependency appears to be less pronounced in semi-crystalline PLA, although a broader temperature range would need to be examined for a definitive conclusion.

**Figure 8:**
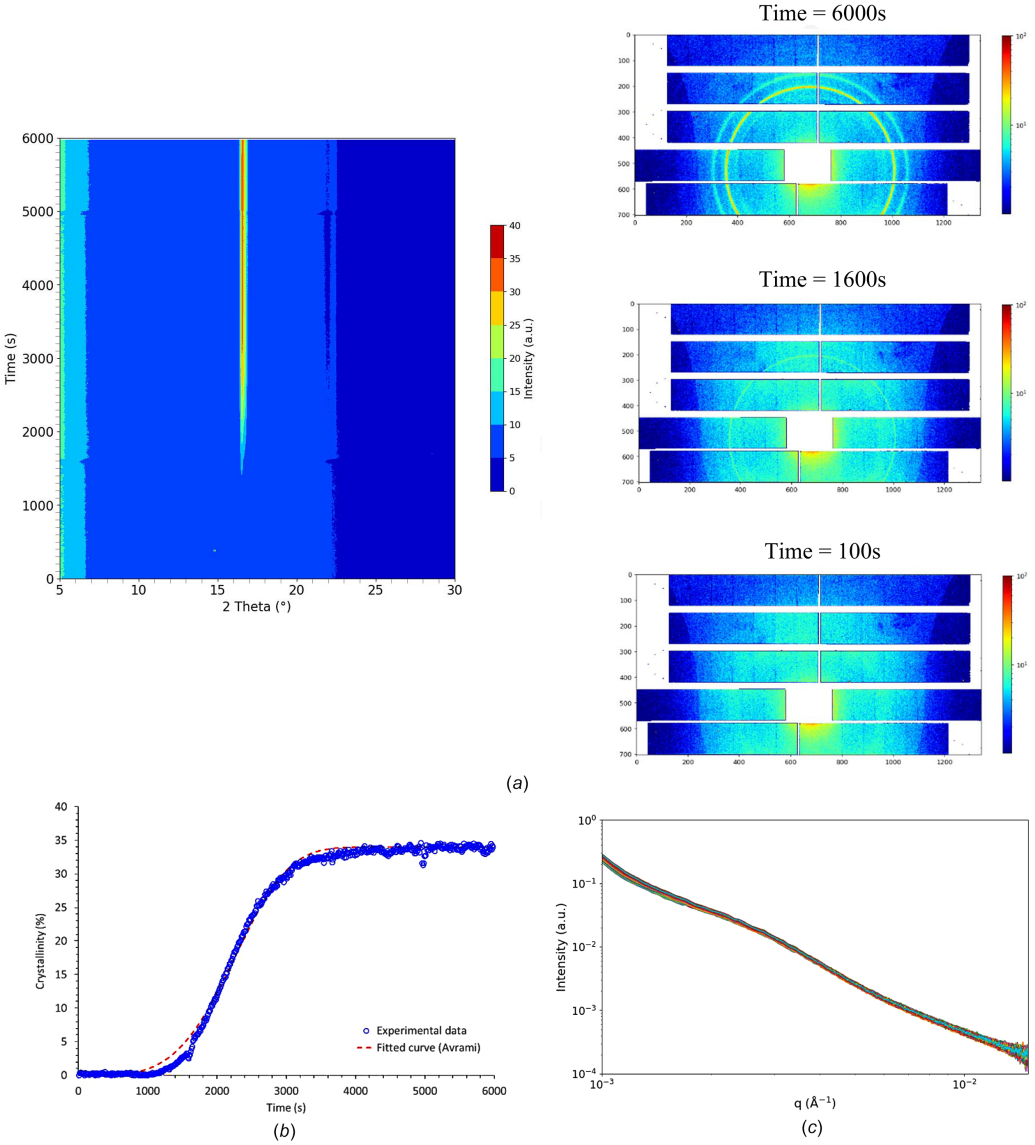
Evolution of (*a*) WAXS scattering and representative WAXS patterns, (*b*) crystallinity and (*c*) the integrated intensity computed from the lambda detector (USAXS) measured during the isothermal crystallization at *T*_c_ = 80°C.

To explore this further and specifically assess the effect of crystallinity on macromolecular network mobility, XPCS analyses were carried out during isothermal crystallization of PLA at *T*_c_ = 80°C. The measurements were carried out on an initially amorphous PLA43D sample and the crystallization temperature was chosen so that the crystallization kinetics would be sufficiently slow, allowing for the accurate measurements of the relaxation times. It is noteworthy that, in this case, crystallization occurred from the solid state and not from the molten state, as in the previous case. Fig. 8 ▸ depicts the structural evolution of the sample during isothermal crystallization from the solid state at *T*_c_ = 80°C.

As shown in Figs. 8 ▸(*a*) and 8(*b*), crystallization begins after ∼1500 s and is completed after ∼3500 s. The crystalline structure formed is the α′ form of PLA (Zhang *et al.*, 2008 ▸), as expected from the literature. The evolution of the crystallinity degree over time, calculated by deconvolution of the WAXS profiles according to the methodology proposed by Stoclet *et al.* (2010*a* ▸), is depicted in Fig. 8 ▸(*b*). In order to obtain information about the induced crystalline morphology, the kinetics were fitted with Avrami’s law expressed as follows,
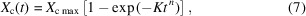
where *X*_cmax_ stands for the maximum crystallinity degree achieved by the material, *K* is a constant related to the crystallization kinetics and *n* is the Avrami exponent.

The crystallization kinetics exhibit a classical sigmoidal shape and the fit leads to values of 2.5 × 10^−15^ s^−1^ for *K* and 4.3, *i.e* close to 4, for the Avrami’s exponent *n*. The latter value is characteristic of a spherulitic growth from sporadic nuclei. From a microstructural point of view, these data suggest that the silica nanoparticles, which have a diameter larger than the amorphous layers, cannot be located between the crystalline lamellae, due to the relatively small inter-lamellae spacing. As a result, they are unable to penetrate into the spherulites and are instead localized in the amorphous regions between the spherulites, *i.e.* in the inter-spherulitic amorphous zones.

**Figure 9:**
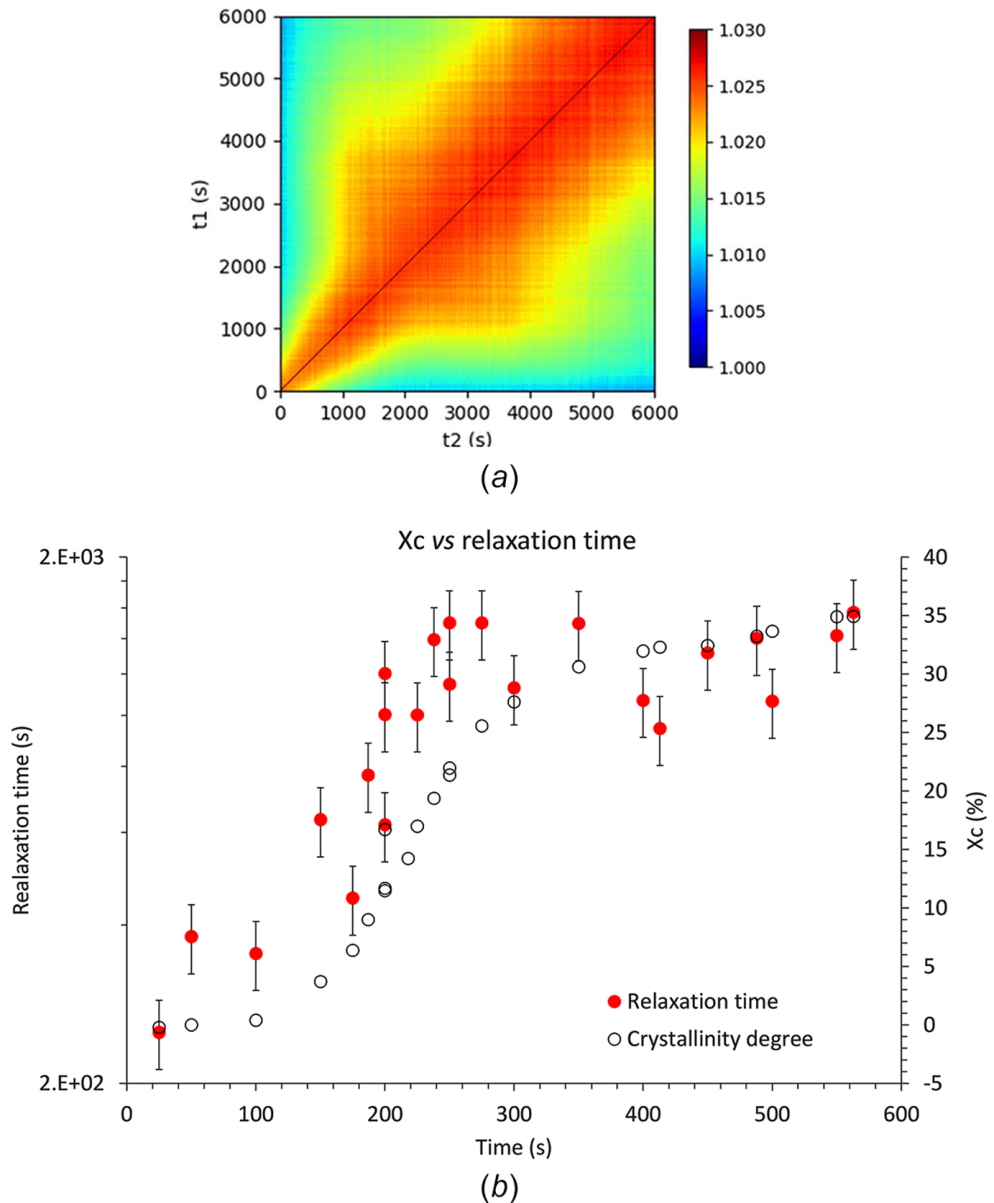
(*a*) Two times correlation function computed from the isothermal measurement and (*b*) evolution of the relaxation time as a function of the crystallinity degree, measured *in situ* for *T*_c_ = 80°C at *q* = 0.01 Å^−1^.

To analyse the data from a relaxation time perspective, the XPCS patterns were processed in batches of 100 images. Using this approach, each batch corresponds to an average crystallinity degree (calculated from the deconvolution of the WAXS averaged patterns). Fig. 9 ▸ depicts the two-time correlation function calculated from the overall measurements during the isothermal crystallization of the PLA43D sample (initially amorphous) at *T* = 80°C, as well as the evolution of the relaxation time τ as a function of the crystallinity degree.

The two-time correlation function presented in Fig. 9 ▸(*a*) allows a qualitative analysis of the effect of crystallization on the relaxation time τ. A broadening of the signal is observed between 1500 s and 3000 s, indicating changes occurring during crystallization. After the maximum crystallinity is reached, the signal remains constant until the end of the measurement. This broadening of the the two-time correlation function corroborates the previous results, showing that crystallinity induces an increase of the relaxation time, *i.e.* a slow down of the dynamics. Fig. 9 ▸(*b*) offers a quantitative analysis, clearly illustrating the close correlation between the measured relaxation time and the crystallinity degree. Specifically, the relaxation time τ, which is approximately 300 s when the sample is amorphous, steadily increases to reach a value slightly above 1000 s when the crystallinity reaches its maximum level, around 35%. Regarding the origin of the increase of τ with crystallinity, it can be hypothesized that it indicates a constraining effect on the macromolecular network. The confinement effect of the amorphous phase by the crystalline lamellae has been previously reported in the case of PLA (Brás *et al.*, 2008 ▸; Zhang *et al.*, 2019 ▸; Nguyen *et al.*, 2015 ▸). However, those studies, carried out by broadband dielectric spectroscopy of differential scanning calorimetry, provided information at a local scale, either at the molecular level or on the scale of few monomers. Consequently, the confinement effect involved in these studies rather corresponds to the confinement of the amorphous layers trapped between the crystalline lamellae. In contrast, XPCS measurements – at least with the conditions used in this work – provide information at a larger scale. As previously mentioned, it is assumed that the nanoparticles are located between the spherulites into the materials. Therefore the observed increase in relaxation times gives insight into the confinement effect, which is not by the crystalline lamellae themselves but rather by the spherulites on the macromolecular network. Thus, XPCS proves to be a powerful tool for probing the effect of parameters such as crystallinity on the macromolecular network dynamics and one can imagine that, by varying the size of the filler, it could be possible to probe the dynamics at different length scales.

However, it is important to note that an effect related to the increase in the concentration of silica nanoparticles within the amorphous domains – driven by the formation of crystalline domains – cannot be entirely excluded. Nevertheless, no changes were observed in the USAXS signal at low *q* (Fig. 8 ▸*c*), suggesting that the dispersion of the silica nanoparticles remains relatively unchanged during the measurements. While the decrease in the amorphous fraction from 100% to 65% corresponds to an increase in the weight fraction of silica nanoparticles in the amorphous domains from 10 wt% to 15 wt%, the USAXS scattering curves in the low *q* region, which are specifically associated with scattering from the silica nanoparticles, remain constant. This indicates that this increase in nanoparticle concentration does not involve inter-particle interactions.

Another interesting finding highlighted by these measurements is that the relaxation time measured depends not only on the crystallinity degree but also on the crystalline morphology into the material. Indeed, even if the samples studied after complete crystallization at 120°C or during isothermal crystallization at *T*_c_ = 80°C exhibit the same crystallinity degrees, their relaxation times differ significantly (*i.e.* 6000 s versus ∼1000 s). This discrepancy can be attributed to differences in crystalline morphology between the two samples, which are directly influenced by the crystallization conditions (Pluta & Galeski, 2002 ▸; Fernandes Nassar *et al.*, 2017 ▸).

### 6.4. Influence of stretching on the polymer dynamics

The final part of this study presents preliminary experiments aimed at investigating the potential role of macromolecular orientation on the dynamics of the macromolecular network. The sample studied consists of PLA60 sample filled with 10% of silica nanoparticles, and submitted to a stretching at a drawing temperature *T*_d_ of 65°C using an initial stretching rate (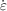) of 10^−2^ s^−1^ up to a draw ratio (ε) of 150%. For the measurements, the stretching axis is placed vertically. Due to the fully amorphous and non-crystallizable PLA grade used, no ordered phase (*e.g.* mesormophic or crystalline) is induced upon stretching. This is further confirmed by the WAXS data, which indicate that the sample only consists of an oriented amorphous phase. In terms of the measurement procedure, the stretched sample was fixed with glue on a sample holder in order to maintain a constant length and minimize its relaxation during heating. The analysis temperature of 65°C was selected to ensure a relatively fast relaxation time with a limited macromolecular relaxation during the measurement. The measurement consists of 400 successive frames with an acquisition time of 10 s per frame (overall measurement duration ∼1 h).

**Figure 10:**
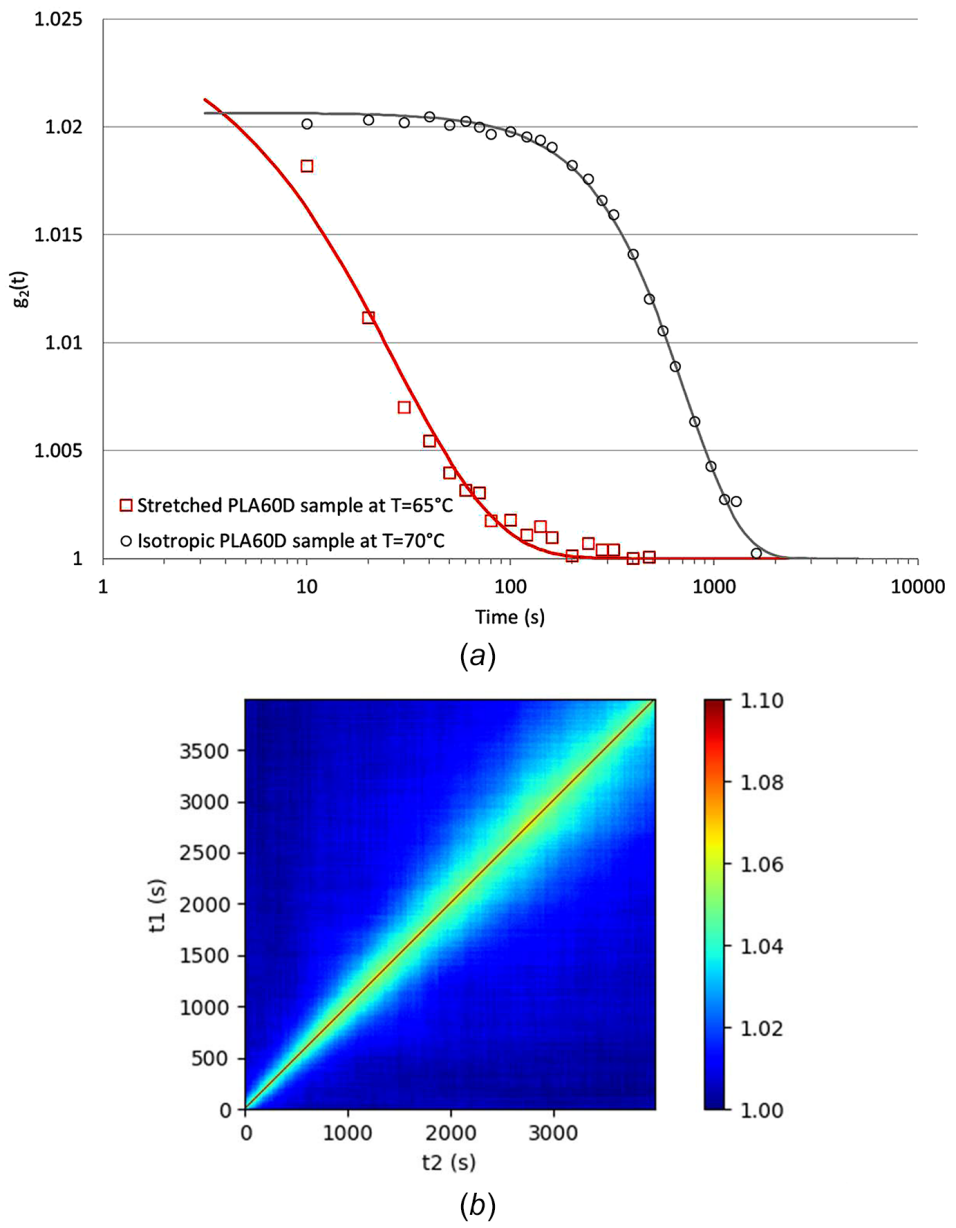
(*a*) Comparison of the autocorrelation functions obtained for the stretched sample at *T* = 65°C and for an isotropic sample at *T* = 70°C and (*b*) two times correlation function obtained for the stretched sample.

Fig. 10 ▸(*a*) clearly demonstrates that macromolecular orientation results in a significant decrease in the relaxation time τ. Specifically, while the relaxation time at this temperature is ∼1000 s in the case of the isotropic sample, it drops by nearly two orders of magnitude, reaching the tens of seconds range in the case of the oriented sample. Another notable feature is the gradual increase in the relaxation time observed during the measurement, as shown by the two-time correlation function depicted in Fig. 10 ▸(*b*). This increase can be interpreted as a result of the sample’s macromolecular mobility at 65°C, which allows it to undergo structural relaxation, leading to a slow reduction in the degree of orientation. This behaviour has not been reported in stretched elastomer (Ehrburger-Dolle *et al.*, 2012 ▸; Ehrburger-Dolle *et al.*, 2019 ▸); these materials, being cross-linked, are less susceptible to such relaxation effects. A dependence of the relaxation time with the orientation degree has already been measured at the molecular scale and reported in the case of PET (Duchesne *et al.*, 2002 ▸; Oultache *et al.*, 2001 ▸). This result, and more generally determining the effect of macromolecular orientation on the dynamics, is of prime interest as previous works carried out on PLA (Stoclet *et al.*, 2010*a* ▸; Stoclet *et al.*, 2010*b* ▸) or PET (Mulligan & Cakmak, 2005 ▸; Blundell *et al.*, 2000 ▸; Mahendrasingam *et al.*, 2005 ▸), suggested that the nature of the strain-induced structure, *i.e.* crystalline, mesomorphic, *etc*., is linked to the molecular dynamics and the macromolecular mobility into the material. Nevertheless, this assumption is based on relaxation times measurements of isotropic samples and the effect of orientation has not yet been clearly established. This example demonstrates that XPCS can be a powerful and appropriate tool for measuring *in situ* the effect of orientation and stretching on the material’s dynamics.

## 7. Conclusion and outlooks

From an experimental perspective, this work demonstrates the feasibility of conducting XPCS measurements on the BM02-D2AM beamline, marking one of the few examples of XPCS on a beamline with a bending magnet source (Cai *et al.*, 1994 ▸). BM02-D2AM is particularly well suited for studying slow dynamics, with relaxation times of tens of seconds or more, on samples sensitive to radiation damage. The ability to simultaneously track the structural evolution through WAXS clearly provides added value. Future upgrades to the beamline, including new optics and detectors, are expected to further enhance the capabilities of XPCS measurements.

From a scientific standpoint this work tackles the influence of various parameters on the dynamics of entangled polymers. Specifically, it was shown that the evolution of the relaxation time with temperature is closely linked to the material’s viscoelastic properties. This highlights the suitability of XPCS for tracking macromolecular network dynamics.

Moreover, for the first time, the influence of crystallinity on dynamics was explored. The results indicate that increasing crystallinity leads to a slowdown in macromolecular network dynamics. Additionally, the impact of crystalline morphology on dynamics was emphasized, opening new avenues for research, though further studies are needed for a comprehensive understanding.

Lastly, the effect of macromolecular orientation on polymer dynamics was assessed. Similar to elastomers, macromolecular orientation was found to induce anisotropic dynamics in the sample. Notably, stretching was shown to accelerate dynamics compared with the isotropic sample. These findings are of great interest, as they contribute to a deeper understanding of the strain-induced structural evolution of polymers during stretching, which is closely linked to macromolecular relaxation processes.

## Supplementary Material

Supporting information. DOI: 10.1107/S1600577525001626/vy5036sup1.pdf

## Figures and Tables

**Table 1:** Characteristics of the PLA grades used in this work (the average molecular weights *M*_n_ and *M*_w_ were determined by size exclusion chromatography and *T*_g_ by means of differential scanning calorimetry)

Grade	*M*_n_ (kg mol^−1^)	*M*_w_ (kg mol^−1^)	*T*_g_ (°C)
PLA60D	61	104	60
PLA43D	75	124	60

**Table 2:** Evolution of the coherence degree of the flux as a function of the primary slits (S1) opening

S1 slits opening (mm)	Flux (photons s^−1^)	Coherence degree (%)	Coherent flux (photons s^−1^)
0.2 × 0.2	2.73 × 10^7^	8.73	2.38 × 10^6^
0.3 × 0.3	6.28 × 10^7^	4.95	3.16 × 10^6^
0.4 × 0.4	1.06 × 10^8^	3.38	3.66 × 10^6^
**0.5 × 0.5**	**1.67 × 10^8^**	**2.52**	**4.20 × 10^6^**
0.6 × 0.6	2.41 × 10^8^	1.76	4.24 × 10^6^
0.7 × 0.7	3.27 × 10^8^	1.32	4.32 × 10^6^
0.8 × 0.8	4.19 × 10^8^	1.04	4.39 × 10^6^
