# Supplementary material for: Structural dependency of polymer dynamics by means of small-angle X-ray photon correlation spectroscopy and wide-angle X-ray scattering on the D2AM beamline
Source: J Synchrotron Radiat. 2025 Apr 1;32(Pt 3):649–60. doi: 10.1107/S1600577525001626 (PMC12067334; doi:10.1107/S1600577525001626)
Supplement: Supplementary file 1 [file s-32-00649-sup1.pdf]

JOURNAL OF SYNCHROTRON RADIATION

Volume 32 (2025)

Supporting information for article:

Structural dependency of polymer dynamics by means of Small Angle X-ray Photon Correlation Spectroscopy and Wide-Angle X-ray Scattering on the D2AM beamline

Gregory Stoclet, Duncan Schwaller, Romain Garlet, Frederic Livet, Gilbert A. Chahine, Nils Blanc and Maxime Dupraz

S1. WAXS and SAXS Analyses of the samples

Prior to the XPCS measurements, the structural characterization of the samples has been carried out by means of SAXS and WAXS. Figure SI-1 depicts the results obtained for both PLA60D and PLA43D filled with 10wt% of silica nanoparticles.

(a) (b)

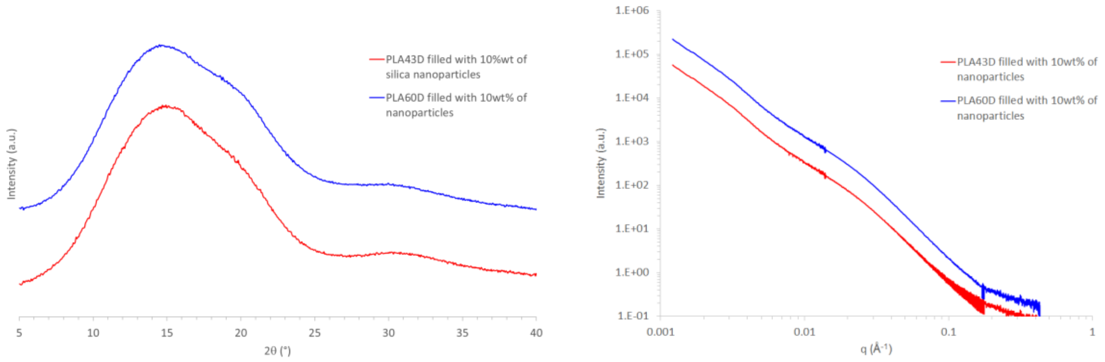

Figure S1 (a) WAXS and (b) SAXS analyses of the as-elaborated films.

The WAXS data depicted in Figure S1(a) indicates that both materials are amorphous after elaboration. This was expected for PLA60D as this material can't crystallize. For PLA43D, which is able to crystallize, this indicates that the cooling rate during the films elaboration was fast enough to prevent crystallization. The SAXS results shown on Figure S1(b) exhibit a strong intensity originating from the scattering from the nanoparticles. The fact that no peak is observed in the investigated q range suggests that the nanoparticles aren't agglomerated and relatively well dispersed into the polymer matrix.

S2. Scanning Electronic Microscopy (SEM) analyses of the samples

SEM analyses have been carried out in order to assess the dispersion degree of the silica nanoparticles into the polymer matrix. Figure S2 depicts representative micrographs of both the raw silica powder and the PLA43D sample filled with 10wt% of nanoparticles. The images were acquired using a Phenom ProX Desktop SEM operating at 10kV.

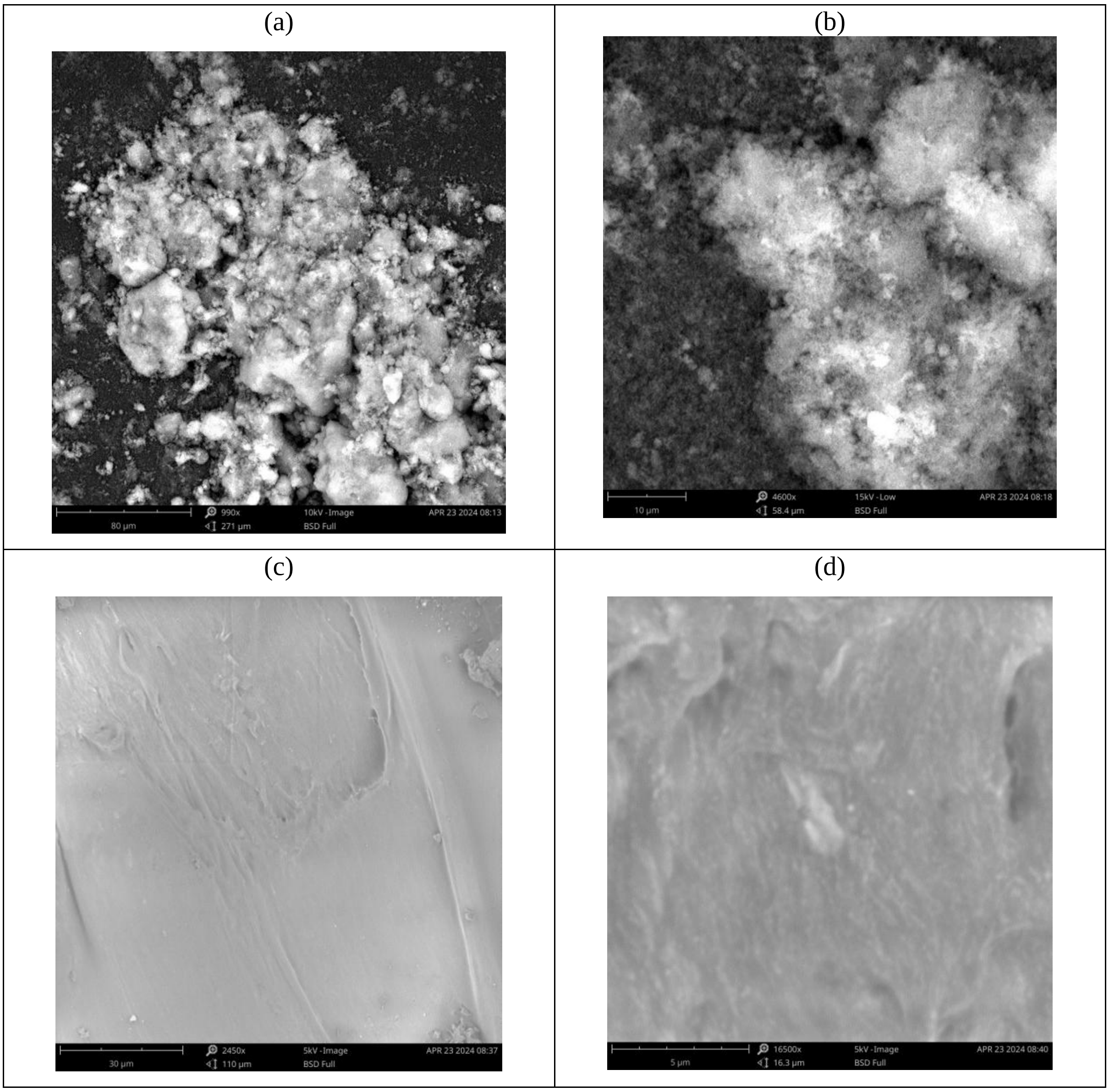

Figure S2 SEM micrographs of the (a-b) raw silica powder and (c-d) PLA43D sample filled with 10%wt of SiO_2 nanoparticles.

The raw silica powder [Figs. S2(a) and S2(b)] consists in micronic agglomerates of the primary silica nanoparticles (mean diameter 200-300nm). Those aggregates are no more visible for the PLA43D sample [Figs. S2(c) and S2(d)] suggesting that (i) those aggregates have been broken during the elaboration of the samples and (ii) that the primary silica nanoparticles are homogeneously dispersed into the polymer matrix.

S3. Beamline and setup characteristics

S3.1. Photon flux on the BM02/D2AM beamline

The BM02 uses a short bending magnet (SBM) with a 0.86T peak field to generate the X-ray beam. As seen from Fig. S3 below, the expected photon flux in 1mrad (H) & 0.5 mrad (V) and for a bandwidth of 0.1% is ~ $2.5\text{x}10^{13}$ ph/s.

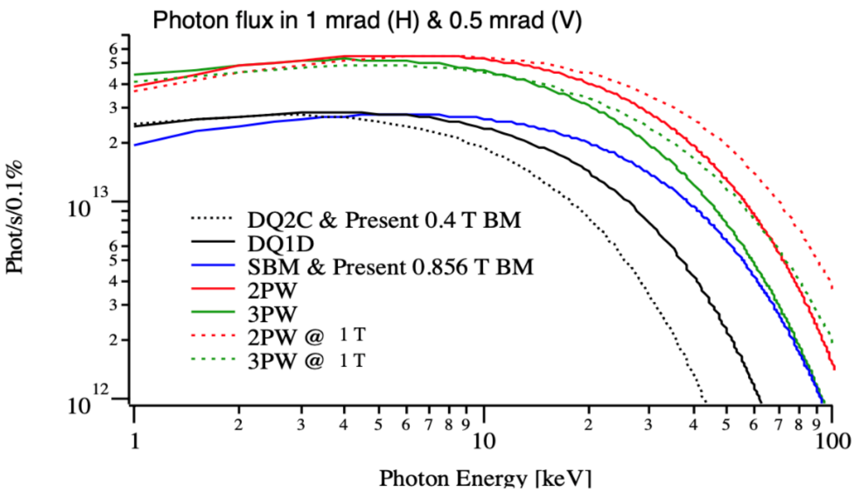

Figure S3 Photon flux as a function of the energy for different magnet elements (BM02 is equipped of a SBM magnet).

The source size and divergence (at 8 keV) are given in Table S1:

Table S1 Source size and divergence on the BM02 beamline at 8 keV.

Source size (V×H), µm	Source divergence (V×H), mrad
8.5×54	0.23×2.04

This gives a beam size at 27.2 meters from the source of 6.2×55.4 mm (V×H). As a reminder, the energy resolution of the Si (111) DCM is of the order of 1.4×10^{-4}. If the S1 slits were fully opened at 6.2×55.4 mm, we can expect a theoretical flux of:

$$Flux_{after\ mono} = Initial\ flux \times \frac{\Delta E_{source}}{\Delta E_{mono}} = 2.5 \times 10^{13} \times \frac{1.4 \times 10^{-4}}{10^{-3}} = 3.5 \times 10^{12}\ ph/s$$

Table S2 Evolution of the flux measured for a 35x35 microns beam at the detector position for different opening of the primary slits and comparison with the theoretical flux.

S1 slits opening (VxH, mm)	Surface slits (mm²)	$I_{measured}$ (ph/s)	$I_{theoritical}$ (ph/s)	$\frac{I_{measured}}{Surface\ slits}$ (ph/mm²)
5×10	50	1.48×10^{11}	5.07×10^{11}	2.96×10^{9}
5×5	25	7.64×10^{10}	2.53×10^{11}	3.06×10^{9}
3×5	15	5.14×10^{10}	1.52×10^{11}	3.43×10^{9}
3×3	9	2.85×10^{10}	9.12×10^{10}	3.17×10^{9}
2×2	4	1.36×10^{10}	4.05×10^{10}	3.40×10^{9}
1×1	1	3.39×10^{9}	1.01×10^{10}	3.39×10^{9}
0.75×0.75	0.5625	1.94×10^{9}	5.70×10^{9}	3.45×10^{9}
0.5×0.5	0.25	8.65×10^{8}	2.53×10^{9}	3.46×10^{9}
0.4×0.4	0.16	5.52×10^{8}	1.62×10^{9}	3.45×10^{9}
0.3×0.3	0.09	3.12×10^{8}	9.12×10^{8}	3.46×10^{9}
0.2×0.2	0.04	1.31×10^{8}	4.05×10^{8}	3.27×10^{9}
0.1×0.1	0.01	3.00×10^{7}	1.01×10^{8}	3.00×10^{9}
0.05×0.05	0.0025	7.38×10^{6}	2.53×10^{7}	2.95×10^{9}

Table S2 summarizes the flux measurements obtained using the Lambda detector for various primary slit openings. Note that measurements were not taken with the slits fully opened. As shown in Table S2, the measured flux for the focused beam is roughly proportional to the primary slit opening. However, the measured flux is significantly lower than theoretical predictions, likely due to losses in the beamline optics as described in the next section.

S3.2. Characterization of the beam coherence

To complete the characterization of the SAXPCS setup, the values of the degrees of coherence β as a function of the S1 slits opening have been calculated and compared to the experimental data. A reasonable estimate for the overall value of β involves expressing the experimental contrast as the product of the beam contrast and the detection contrast:

$$\beta(z) \approx \beta(z1)\beta(z2)$$

where $\beta(z)$ can be calculated by a series expansion in the variable z [*Livet, F. Diffraction with a Coherent X-Ray Beam: Dynamics and Imaging. Acta Crystallogr A Found Crystallogr 2007, 63 (2), 87–107.*]

The contrast of the beam is given by:

$$z1 = \pi\phi_1\phi/(\lambda D)$$

where ϕ_1 is the opening of the primary slits S1, ϕ is the size of the beam at the sample position and D the distance between the sample and the S1 slits

On the detection side:

$$z2 = \pi\phi\delta/(\lambda d)$$

where δ is the detector pixel size and d is the sample detector distance.

For our experimental setup, ϕ_1, λ, and δ are known values (0.5 mm, 1.55 Å, and 55 microns, respectively). Using these parameters, the calculated beam size at the sample is approximately 19 microns, as illustrated in Fig. S4 below:

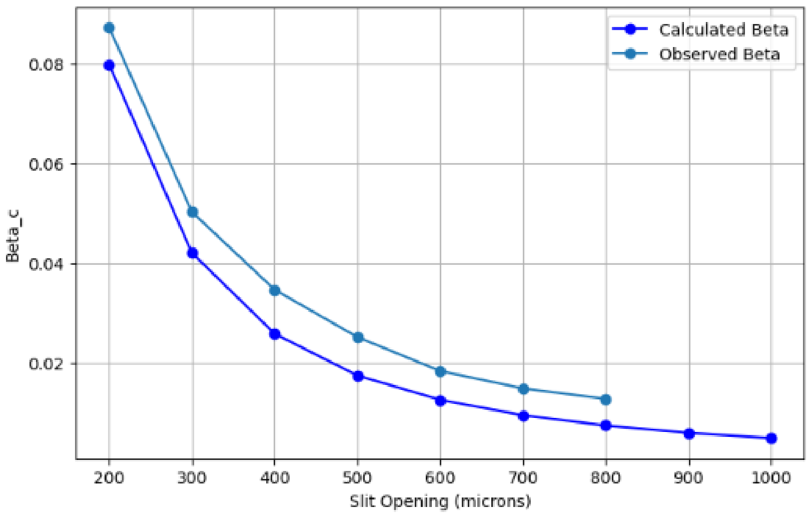

Figure S4 Observed and Calculated β as the function of the S1 slits opening.

S4. Furnace details

Maintaining high stability in the setup is crucial for conducting XPCS measurements. This includes ensuring temperature stability, which is achieved using a high-precision furnace developed by the sample environment support service at ESRF and specifically optimized for these experiments [Fig. S5(a)]. Special attention was given to selecting PID parameters to enable rapid stabilization of the temperature within the chosen range for the experiment. As shown in Fig. S5(b), a stability of better than 0.1°C was attained over the range from room temperature to 180°C. Furthermore, temperature stabilization was relatively quick, typically taking 2-3 minutes, as illustrated in Fig. S5(c).

(a)

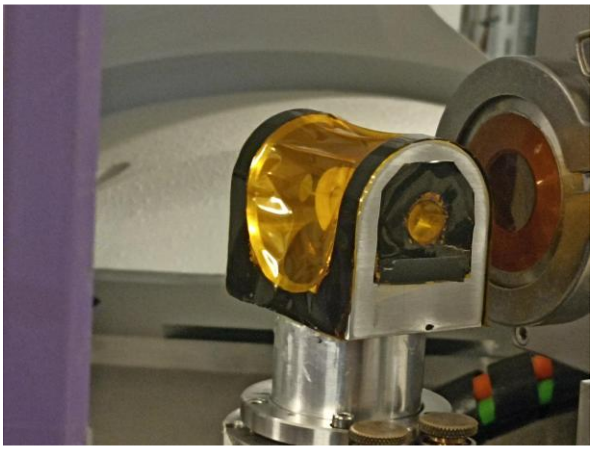

(b)

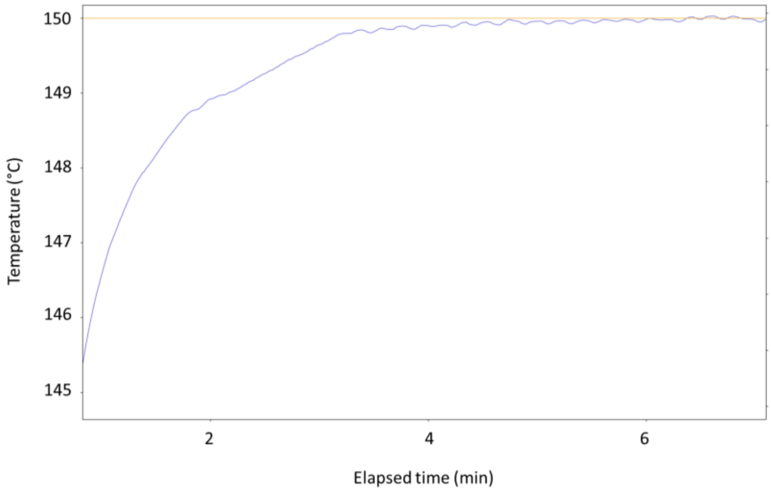

(c)

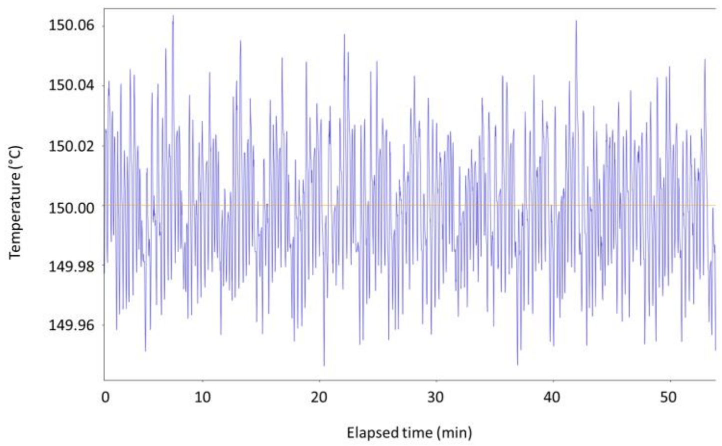

Figure S5 (a) Image of the furnace used. (b) Temperature stabilization at 150°C. (c) Temperature stability at 150°C.
